# Small-Pox

**Published:** 1871-05

**Authors:** 


### SMALL-POX.

The succinct facts in reference to this terrible disease which have been indisputably established by the careful observation of scientific men at home and abroad are, —

1. That infants should be vaccinated in a month after birth.
2. That vaccination should be repeated at the age of fourteen years.
3. That the older persons grow after that, the less danger there is of an attack of small-pox or varioloid, which is small-pox modified by previous vaccination, and that after fifty, there is no danger of an attack.
4. Still, if there is any danger of unusual exposure to the disease, or if it prevails largely in a community, it is safer to be vaccinated again.

5. Nurses in small-pox hospitals who are revaccinated are almost wholly exempt, while other nurses, who are careless of revaccination, have varioloid in cases ten times more numerous.

6. That the farther the remove of the vaccine matter from what was taken from the cow, the more necessary is revaccination; that is, vaccine matter loses some of its power in every person it passes through; hence if city practitioners should take all their matter from the cow direct, every ten years, the community would be greatly safer.

7. No authenticated case has yet occurred where any disease has been communicated through vaccine matter, other than modified small-pox. This statement should be fixed upon the memory of the intelligent reader, as bare assertions to the contrary are recklessly made by a certain class of uneducated persons who write on matters pertaining to health and disease.

8. It is a very rare occurrence that any person who has once been well vaccinated, and was in good health at the time, suffers from varioloid. Those who have varioloid after having been vaccinated once, suffer because it did not take well, or some other disease was in the system at the time, or the matter was at too great a remove from the original source, the cow.

